# Focused ultrasound enhanced molecular imaging and gene therapy for multifusion reporter gene in glioma-bearing rat model

**DOI:** 10.18632/oncotarget.5389

**Published:** 2015-09-25

**Authors:** Feng-Yi Yang, Wen-Yuan Chang, Wei-Ting Lin, Jeng-Jong Hwang, Yi-Chun Chien, Hsin-Ell Wang, Min-Lan Tsai

**Affiliations:** ^1^ Department of Biomedical Imaging and Radiological Sciences, National Yang-Ming University, Taipei, Taiwan; ^2^ Biophotonics and Molecular Imaging Research Center, National Yang-Ming University, Taipei, Taiwan; ^3^ Department of Pediatrics, Cheng Hsin General Hospital, Taipei, Taiwan

**Keywords:** focused ultrasound, molecular imaging, gene therapy, blood-brain barrier, brain tumor

## Abstract

The ability to monitor the responses of and inhibit the growth of brain tumors during gene therapy has been severely limited due to the blood-brain barrier (BBB). A previous study has demonstrated the feasibility of noninvasive *in vivo* imaging with ^123^I-2′-fluoro-2′-deoxy-5-iodo-1-β-D-arabinofuranosyluracil (^123^I-FIAU) for monitoring herpes simplex virus type 1 thymidine kinase (HSV1-tk) cancer gene expression in an experimental animal model. Here, we tested the enhancement of SPECT with ^123^I-FIAU and ganciclovir (GCV) treatment in brain tumors after BBB disruption induced by focused ultrasound (FUS) in the presence of microbubbles. We established an orthotopic F98 glioma-bearing rat model with trifusion reporter genes. The results of this study showed that the rat model of HSV1-tk-expressing glioma cells could be successfully detected by SPECT imaging after FUS-induced BBB disruption on day 10 after implantation. Compared to the control group, animals receiving the GCV with or without sonication exhibited a significant antitumor activity (*P* < 0.05) of glioma cells on day 16 after implantation. Moreover, combining sonication with GCV significantly inhibited tumor growth compared with GCV alone. This study demonstrated that FUS may be used to deliver a wide variety of theranostic agents to the brain for molecular imaging and gene therapy in brain diseases.

## INTRODUCTION

The systemic administration of conventional radiotherapy and chemotherapy can lead to side effects on patients [1, 2]. In contrast with traditional treatments, gene therapy has the potential to be more tumor-specific in terms of targeting and selectively destroying tumor cells by inserting genes conferring drug sensitivity into the tumor cells. Antitumor suicide gene therapy is a new therapeutic strategy for the treatment of patients with otherwise incurable malignant brain tumors. This approach involves the introduction into tumor cells of a gene capable of converting a nontoxic prodrug into a cytotoxic drug. Transfer of the herpes simplex virus type 1 thymidine kinase (HSV1-tk) gene into tumor cells makes these cells sensitive to antiviral drugs [3]. The most frequently used genetic prodrug activation system is the HSV1-tk/ganciclovir (GCV) paradigm. HSV1-tk phosphorylates GCV to monophosphate and then, by cellular kinase, to diphosphate- and triphosphate-GCV. The HSV1-tk/GCV strategy leads to the death of not only the transfected tumor cells but also of surrounding nontransfected tumor cells [4].

Several reporter genes express proteins that can generate signals for *in vivo* imaging, such as the firefly luciferase (Fluc) gene, the green fluorescence protein (GFP) gene, and the HSV1-tk gene [5, 6]. Fluc/GFP/HSV1-tk (FGT) is a triple reporter protein used for multimodal *in vivo* imaging of gene expression with bioluminescence or fluorescence and single photon emission computed tomography (SPECT) imaging [7]. Multimodality imaging approaches allow for various imaging types to be combined during the course of the same study. Noninvasive nuclear imaging can offer information regarding the location and the level of gene expression when an appropriate reporter gene is constructed in the tumor cells [8]. The HSV1-tk gene can be used as both a reporter gene and a therapeutic gene for noninvasive imaging of the gene expression. It has been shown that SPECT imaging with ^123^I-2′-fluoro-2′-deoxy-5-iodo-1-β-D-arabinofuranosyluracil (^123^I-FIAU) is a reliable method for predicting tumor response to GCV treatment, which was found to be proportional to the magnitude of HSV1-tk expression in tumor tissue [9].

Noninvasive monitoring of the distribution of transgene expression over time is critical for the safety and evaluation of the efficacy of gene therapy in clinical applications. Organ dosimetry assessment in rats injected with diagnostic doses of radiolabeled FIAU has revealed very low values for radioactivity within the brain, suggesting limited blood-brain barrier (BBB) penetration [10]. Evaluations of nuclear imaging have shown, however, that focused ultrasound (FUS) with microbubbles not only significantly increases the permeability of the BBB at the sonicated site, but also significantly elevates the tumor-to-normal brain drug ratio in the focal region [11–13]. To further evaluate the applicability of ^123^I-FIAU as a potential marker substrate to image HSV1-tk gene expression in the brain with enhancement of its penetration of the BBB, we used FUS to disrupt the BBB in F98/FGT glioma-bearing rats.

Despite promising preclinical studies, the first clinical trials for treating brain tumors with the HSV1-tk/GCV approach showed discouraging therapeutic effects [14]. In addition, such clinical studies have revealed that while the HSV1-tk/GCV treatment is safe, responses are observed only in very small brain tumors, indicating insufficient vector distribution and very low transduction efficiency [14, 15]. Our own previous study showed that FUS-induced BBB disruption is able to concentrate high-dose chemotherapeutic drugs into brain tumors and improve their antitumor effects [16]. It has also been demonstrated that non-viral delivery can be enhanced by FUS-induced BBB disruption [17]. To elevate treatment efficacy, techniques for enhancing the delivery and distribution of therapeutic genes will need to be investigated. In the present study, we demonstrate that both molecular imaging and gene therapy can be enhanced by FUS in the presence of microbubbles in the F98/FGT glioma-bearing rat model.

## RESULTS

**Figure 1:**
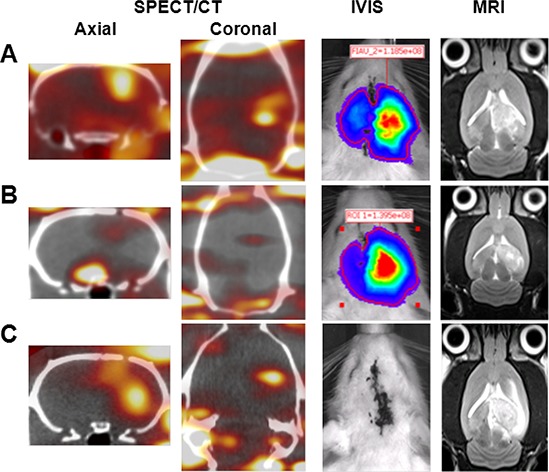
Representative micro-SPECT/CT, bioluminescence, and MR images obtained on day 10 after tumor implantation F98/FGT gliomas with and without FUS-induced BBB disruption are shown in **A.** and **B.** respectively. F98 gliomas with BBB disruption are shown in **C.**

The main aim of this study was to determine whether the molecular imaging of an established intracranial brain tumor derived from F98/FGT glioma cells with ^123^I-FIAU could be enhanced by pulsed FUS exposure. The efficacy of noninvasively monitoring the sites of development of F98/FGT gliomas was assessed by micro-SPECT/CT with ^123^I-FIAU (Figure 1). In addition, the corresponding bioluminescence and MRI images were observed for tumor cell progression and size evaluation. High levels of ^123^I-FIAU radioactivity accumulation in F98/FGT gliomas with FUS-induced BBB disruption revealed a high level of HSV1-tk expression (Figure 1A). In contrast, only low uptake was found in the F98/FGT control group (Figure 1B). Furthermore, the accumulation of ^123^I-FIAU was higher in F98 gliomas with FUS-induced BBB disruption than in the F98/FGT control group (Figure 1C). Additionally, the F98 gliomas (Fluc negative) showed no luciferase activity.

**Figure 2:**
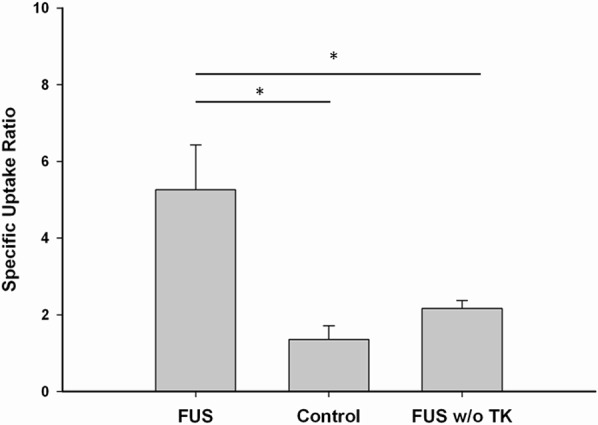
Specific uptake ratio of ^123^I-FIAU derived from micro-SPECT/CT images Each group represents mean ± SEM of 6 rats. (*, *p* < 0.05)

The SUR of ^123^I-FIAU in the F98/FGT gliomas with or without sonication and in the F98 gliomas with sonication derived from microSPECT/CT images are shown in Figure 2. Among these three groups, a significantly highest value of SUR can be seen in the F98/FGT gliomas with FUS-induced BBB disruption. However, there was a slight but insignificant increase in the SUR of ^123^I-FIAU in the F98 gliomas (HSV1-tk negative) with sonication compared with the control group.

**Figure 3:**
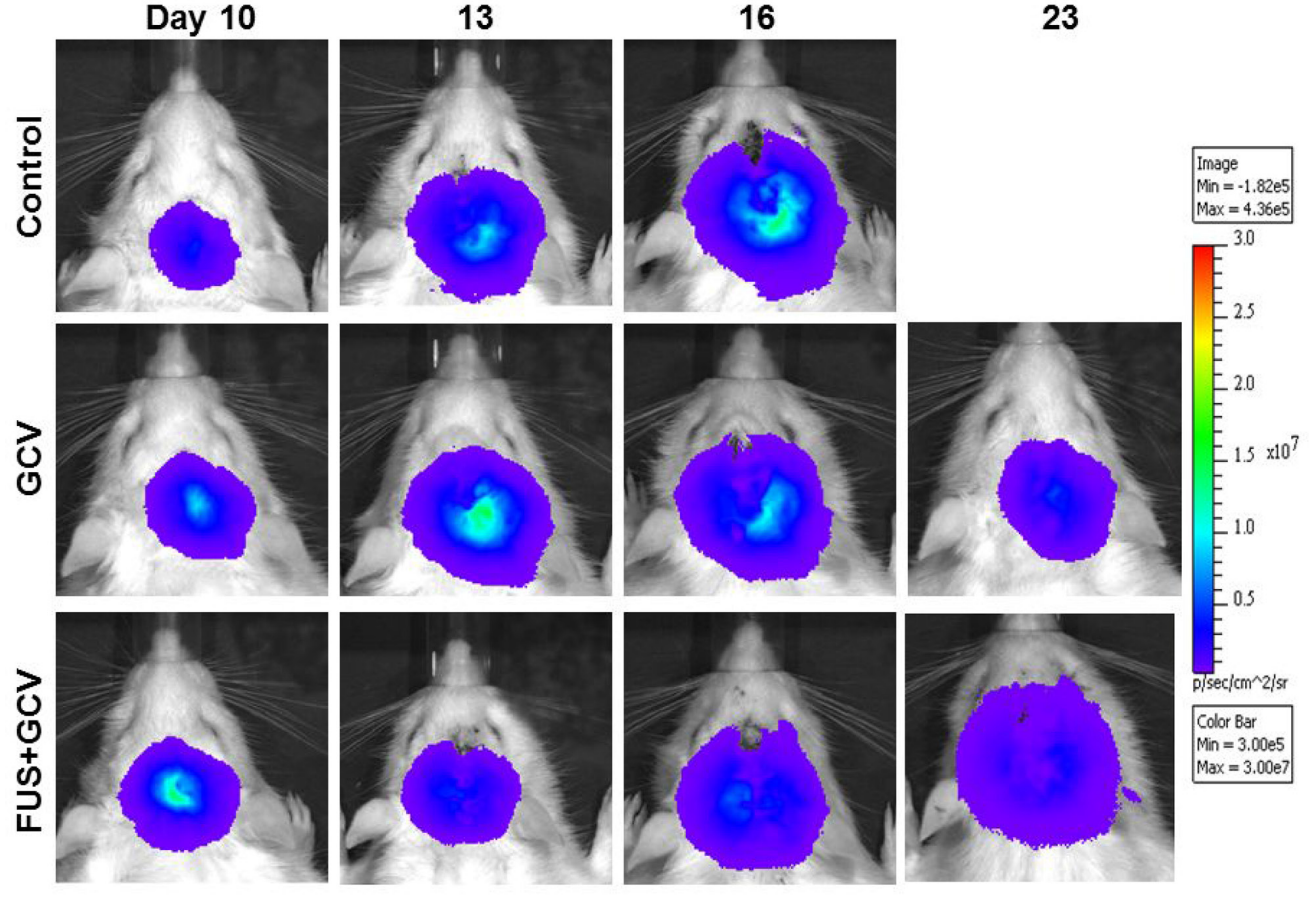
Biophotonic imaging of longitudinal brain tumor monitoring from days 10 to 23 after implantation Trifusion reporter-expressing F98 glioma cells were implanted into the right brain of Fischer 344 rats and received no treatment (control), treatment with GCV from days 11 to 17, or treatment with pulsed FUS before GCV injection on days 11, 14, and 17.

To determine the antitumor effects, tumor-bearing rats were treated with various protocols from days 11 to 17, and tumor progressions were evaluated by IVIS imaging over time (Figure 3). Tumor cells spread rapidly and all rats in the untreated control group expired before day 23 (Figure 3, top panel). Inhibition of tumor cell growth was clear with GCV treatment, but was even more marked for rats treated with GCV followed by pulsed FUS (Figure 3, bottom panel).

**Figure 4:**
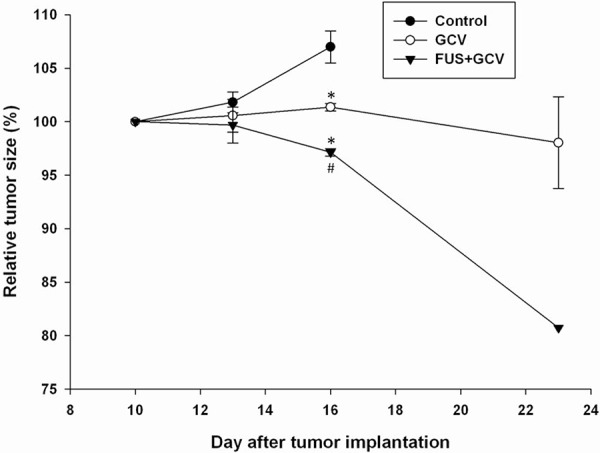
Analysis of increases in tumor size (relative to day 10) based on data obtained from the biophotonic images in Figure 5 * and ^#^ denote significant differences compared to the control group and the group that received GCV alone, respectively. (*,^#^*P* < 0.05, *n* = 3, triplicate in each experiment).

Treatment of the tumors with GCV with or without FUS exposure significantly slowed the growth of the tumor on day 16 after implantation (Figure 4). Moreover, a significant improvement was observed in the antitumor effect in rats treated with GCV plus sonication compared to GCV alone on day 16 after implantation.

## DISCUSSION

The main purpose of this study was to evaluate whether molecular imaging and gene therapy can be improved by FUS-induced BBB disruption in the F98/FGT glioma-bearing rat model. These experiments demonstrated that FUS can enhance the delivery of ^123^I-FIAU and GCV for the HSV1-tk gene-expressing tumor cells in an F98 brain tumor model.

In the current study, the advantages of multi-modality imaging using the Fluc/HSV1-tk fusion reporter gene are well established. Firstly, MR imaging is used to accurately identify the location of a tumor in brain tissue. Furthermore, the Fluc subunit provides for bioluminescence imaging, and the HSV1-tk subunit provides nuclear imaging capabilities (Figure 1). This fusion protein is useful as a tool for quickly validating various approaches in small animal models that can be rapidly translated to clinical applications.

Despite the fact that the blood-tumor barrier (BTB) is itself more permeable than the BBB, the efficacy of systemic chemotherapy is poor in patients with brain tumors because the selective permeability of the BTB still restricts the delivery of drugs into the tumor [18]. Our results indicate that FIAU does not penetrate the BBB in the HSV1-tk gene-expressing glioma cells sufficiently, whereas significant FIAU accumulation occurs in the HSV1-tk gene-expressing glioma cells with FUS-induced BBB disruption (Figure 2). The limited BBB permeability of ^123^I-FIAU is consistent with the occurrence of no significant FIAU uptake in normal brain tissue [19]. Thus, ^123^I-FIAU may not be an appropriate marker substrate for the noninvasive localization of HSV1-tk gene expression in the brain under conditions in which the permeability of the BBB is likely to be limited. In addition, a slight but non-significant increase was found in the SUR of ^123^I-FIAU in the F98 glioma cells (HSV1-tk negative) with FUS-induced BBB disruption compared with the control group of HSV1-tk-positive glioma cells. This phenomenon suggests that the integration of marker substrate of the HSV1-tk enzyme and FUS technology represents a synergistic approach for enhanced molecular imaging in brain tumors.

Gene therapy with the HSV1-tk suicide gene in combination with the prodrug GCV has the potential to be an alternative approach for battling brain tumors [14, 20]. A successful regression of HSV1-tk transfected brain tumors in response to GCV has been well documented in rats [20, 21]. However, the major obstacles for brain tumor treatment with this method are poor delivery of GCV and the insufficient amounts of the suicide gene transfected into the target tumor tissue. Association of GCV with a BTB modifier such as the bradykinin agonist RMP-7 could significantly enhance the uptake of GCV across the BTB and subsequently eliminate the persisting transduced cells [22]. The present findings indicate that FUS may enhance the tumoricidal effect of GCV by selectively increasing the permeability of the BBB in the tumor site (Figures 3 and 4). Hence, when administered in combination with sonication, GCV may reach higher concentrations within the tumor tissue that could enhance its cytocidal effect in transduced HSV1-tk glioma cells. Increased BTB permeability to other nuclear imaging probes or chemotherapeutics by FUS has been demonstrated [11–13, 16]. It has been shown that the possible mechanism involved is the opening of the tight junctions between microvascular endothelial cells in the brain [23].

Previous and present studies have confirmed that the delivery of molecular imaging agents and the efficacy of HSV1-tk/GCV gene therapy can be improved with the enhancement of BBB permeability [22, 24]. Moreover, the use of FUS may help to ameliorate its cytotoxic side effect by a reduction in the systemic dose of GCV. The results of the present study indicate that FUS could be an important adjunctive treatment for noninvasive gene transfer imaging and gene therapy in brain diseases.

## MATERIALS AND METHODS

### Cell culture and transduction

The F98 rat glioma cells, a generous gift from Dr. Rolf F. Barth (Department of Pathology, the Ohio State University, USA), were cultured in Dulbecco's modified Eagle's medium (DMEM) containing 10% FBS and 1% penicillin/streptomycin (Gibco) and maintained in a humidified incubator at 37°C. To establish the F98/FGT stable clone, 3 × 10^6^ F98 cells were suspended in 1 ml OptiMEM (Gibco) supplemented with polybrene (8 μg/ml) and p*Fu*-FGT lentiviral vector (a kind gift from Dr. Sanjiv S. Gambhir, Stanford University, USA) with varying MOIs (multiplicities of infection) in a T75 flask. After 4 hr incubation, DMEM was added up to 15 ml to stop the transduction. Lentivirus-containing medium was replaced by fresh DMEM at 24 hr after transduction. To obtain the F98/FGT stable clone, the transduced F98 cells (10^7^ cells/ml) were sorted with an FACSCalibur flow cytometer (BD Biosciences), and the parental F98 cells were used to set the gating threshold (GFP expression). 6 × 10^5^ sorted GFP-positive F98/FGT cells were maintained in a T75 flask and sub-cultured up to tenth passages. The GFP expression was monitored by flow cytometry at every passage. The clone stably expresses GFP was renamed as F98/FGT cell line.

### Triple-fusion reporter gene in glioma model

**Figure 5:**
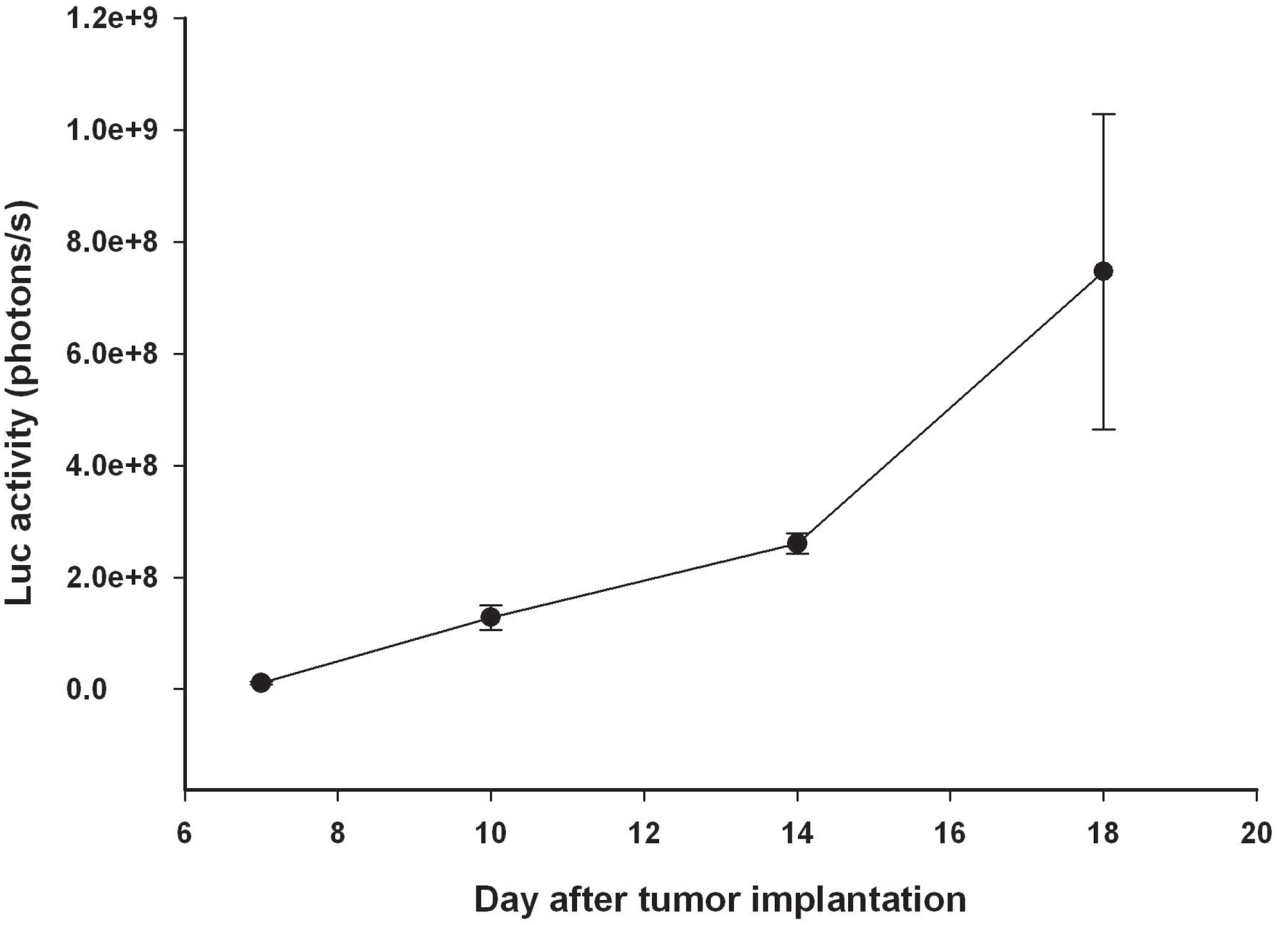
Tumor progression in F98/FGT glioma-bearing rats was monitored by bioluminescence images Each point consisted of three rats.

In this study, we used lentivirus vectors constructed with trifusion reporter genes (Fluc/GFP/HSV1-tk) and transduced into rat F98 glioma cells to establish an orthotopic F98/FGT glioma-bearing rat model. Male Fischer 344 rats (11–13 weeks, approximately 250–280 g) were anesthetized with an intraperitoneal administration of pentobarbital at a dose of 40 mg/kg of body weight. Then 1 × 10^5^ F98/FGT rat glioma cells in 10 μL Hanks’ balanced salt solution without Mg^2+^ and Ca^2+^ were stereotactically injected into the right hemisphere (5.0 mm posterior and 3.0 mm lateral to the bregma) of each rat at a depth of 5.0 mm from the brain surface. Next, the holes in the skull were sealed with bone wax and the wound was flushed with iodinated alcohol. To assess the F98/FGT glioma-bearing rat model, tumor size was quantified by analysis of their biophotonic images obtained from 7 days to 18 days after tumor implantation (Figure 5). All procedures involving animals were in accordance with the guidelines for the Care and Use of Laboratory Animals. This study protocol was approved by Animal Care and Use Committee of National Yang Ming University.

### Synthesis of ^123^I-FIAU

FIAU was purchased from Sigma. GCV was purchased from Roche, Inc. ^123^I-FIAU was synthesized from 5-tributylstannyl-2′-fluoro-1-β-D-arabinofuranosyluracil (FTAU) [25, 26]. The radiochemical purity of ^123^I-FIAU was determined using radio-thin-layer chromatography (TLC). Radio-TLC was performed on an aluminum sheet coated with silica gel powder (Silica gel 60, 70–230 mesh; Merck) by using ethyl acetate/ethanol = 90:10 (v/v) as the mobile phase. The final product, lyophilized ^123^I-FIAU, was redissolved in ethanol, and the radiochemical purity was determined using radio-TLC, which showed the R_f_ of ^123^I-FIAU to be 0.82–0.83, which is the same value that would be obtained from an authentic FIAU standard. The labeling yield was >95%, and the radiochemical purity was >98%.

### Pulsed FUS and the treatment protocol

Pulsed FUS exposures were generated by a 1.0-MHz, single-element focused transducer (A392S, Panametrics, Waltham, MA, USA) with a diameter of 38 mm and a radius of curvature of 63.5 mm. The half-maximum of the pressure amplitude of the focal zone had a diameter and length of 3 mm and 26 mm, respectively. The experimental ultrasound setup was the same as used in our previous study [27, 28]. UCA (SonoVue, Bracco International, Amsterdam, the Netherlands) was injected into the tail vein of the rats approximately 15 s before each sonication. The sonication was precisely targeted using a stereotaxic apparatus (Stoelting, Wood Dale, IL, USA) that utilized the bregma of the skull as an anatomical landmark. Glioma-bearing rats were sonicated at the tumor region on day 10 after tumor cell implantation. The focal zone of the ultrasound beam was delivered to one location in the right brain hemisphere centered on the tumor injection site. Ultrasound transmission gel (Pharmaceutical Innovations, Newark, NJ, USA) was used to cover the area between the transducer and the rat's skull in order to maximize the transmission of the ultrasound. The sonication parameters were as follows: an acoustic power of 2.86 W (corresponding to a peak negative pressure of 0.7 MPa) with an injection of 300 μl/kg UCA, a pulse repetition frequency of 1 Hz, a duty cycle of 5%, and a sonication time of 60 s.

**Figure 6:**
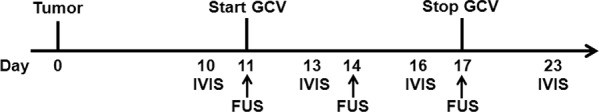
Diagram of the experimental time lines for GCV treatment FUS sonication was applied on days 11, 14, and 17 after tumor implantation. F98/FGT glioma-bearing rats received intraperitoneal administration of GCV daily for a period of 7 days. GCV: HSV1-tk/ganciclovir, FUS: focused ultrasound, IVIS: Xenogen IVIS 50 imaging.

Two experimental protocols were followed to evaluate, firstly, the enhancement of the molecular imaging following FUS-induced BBB disruption and, secondly, the effect of FUS exposure on the GCV treatment. In the first protocol, twelve F98/FGT glioma-bearing rats were divided into two groups for micro-SPECT/CT imaging assessment. Six F98/FGT glioma-bearing rats were sonicated with pulsed FUS in the presence of UCA for BBB disruption. The other six F98/FGT glioma-bearing rats received no FUS treatment. In a third group, six rats were injected with F98 glioma cells and received no FUS treatment. Another experiment was performed to explore the effect of FUS-induced BBB disruption on the GCV treatment (Figure 6). As a control group, six F98/FGT glioma-bearing rats received no treatment. In a GCV group, six F98/FGT glioma-bearing rats were injected intraperitoneally with GCV (10 mg/kg daily) for 7 consecutive days from the eleventh day after tumor implantation. In an FUS+GCV group, six F98/FGT glioma-bearing rats received GCV treatment for 7 consecutive days and underwent a FUS sonication immediately before GCV administration on days 11, 14, and 17 after tumor implantation.

### Micro-SPECT/CT imaging

A FLEX Triumph™ pre-clinical imaging system (Gamma Medica-Ideas, Inc., Northridge, CA, USA) was used for the small-animal SPECT/CT image acquisition. This system applied circular scanning protocols for both SPECT and CT acquisition, with a translation stage in a variable axial imaging range. The axial field of view (FOV) for CT without stage translation was 61.44 mm. The CT system had a power-adjustable X-ray emitter ranging from 50 to 80 kVp and a microfocus (< 50 μm) tube. The SPECT projection dataset was acquired using three low-energy, high-resolution pinhole collimators with a radius of rotation of 50 mm. The rats were anesthetized by inhalation of isoflurane with oxygen and then scanned by CT using 512 slides for anatomic coregistration; they then underwent a SPECT scanning. Static imaging was performed with 3 groups of rats (6 rats per group) for 60 min at 12 h after intravenous injection of 1 mCi/0.5 mL of ^123^I-FIAU. The image dataset was then reconstructed using the ordered subset expectation maximization (OSEM) algorithm with standard mode parameter as provided by the manufacturer. No scatter or attenuation correction was applied to the reconstructed images. A pinhole SPECT acquisition of a standard amount of radioactivity was performed as a reference for quantification, and decay was corrected using radioactivity counts measured with a γ–counter (VDC-405, Veenstra Instruments, The Netherlands).

The images were viewed and quantified using AMIDE software [29] (free software provided by SourceForge). Spherical regions of interest (ROIs, radius = 2.5 mm) under the skull defect were manually pinpointed at the tumor site and in the same region of the contralateral brain. The image counts within the ROIs were converted to absolute radioactivity using an efficiency factor determined from the reference standard radioactivity. In addition, we used contralateral brain as the reference background. The specific uptake ratio (SUR) of ^123^I-FIAU in the tumor site was calculated by subtracting the mean radioactivity within the ROI in the contralateral brain from the mean radioactivity in the ROI of the tumor site and dividing the result by the mean radioactivity in the background, i.e., (tumor radioactivity - background radioactivity / background radioactivity).

### Bioluminescent imaging

To assess the efficacy of GCV treatment, tumor size was quantified by analyzing bioluminescence images obtained from 10 to 23 days after tumor implantation. Each F98/FGT glioma-bearing rat was injected with 150 mg/kg of freshly prepared luciferin substrate suspended in phosphate-buffered saline (PBS). After anesthetic induction with isoflurane, rats were imaged using the Xenogen IVIS 50 imaging system (Xenogen, Palo Alto, CA, USA) 15 min after the intraperitoneal injection of luciferin, with a 5-min acquisition time in small-bin mode. Luciferase activity was viewed and quantified using LIVINGIMAGE V. 3.1 Software from Xenogen within a ROI that encompassed the head of the rat after administration of luciferin substrate to the anesthetized rat.

### Magnetic resonance imaging

To monitor the tumor progression, magnetic resonance imaging (MRI) was performed using a 3-T MRI system (TRIO 3-T MRI, Siemens MAGNETOM, Germany). The rats were anesthetized with isoflurane mixed with oxygen during the imaging procedure. A loop coil (Loop Flex Coil, approximately 4 cm in diameter) was used for RF reception. Tumor progression was monitored by means of T2-weighted images obtained on day 10 after tumor implantation. The parameters for T2-weighted imaging were as follows: repetition time/echo time = 3500/75 ms, matrix = 125 × 256, field of view = 25 × 43 mm, and section thickness = 1.0 mm. The imaging plane was located across the center of the tumor injection site.

### Statistical analysis

All values are shown as means ± SEM. Statistical analysis was performed using an unpaired Student *t* test. The level of statistical significance was set at *P* ≤ 0.05.

## References

[1] Kaldor JM, Day NE, Pettersson F, Clarke EA, Pedersen D, Mehnert W, Bell J, Host H, Prior P, Karjalainen S (1990). Leukemia following chemotherapy for ovarian cancer. The New England journal of medicine.

[2] Relling MV, Rubnitz JE, Rivera GK, Boyett JM, Hancock ML, Felix CA, Kun LE, Walter AW, Evans WE, Pui CH (1999). High incidence of secondary brain tumours after radiotherapy and antimetabolites. Lancet.

[3] Moolten FL (1986). Tumor chemosensitivity conferred by inserted herpes thymidine kinase genes: paradigm for a prospective cancer control strategy. Cancer research.

[4] Freeman SM, Abboud CN, Whartenby KA, Packman CH, Koeplin DS, Moolten FL, Abraham GN (1993). The “bystander effect”: tumor regression when a fraction of the tumor mass is genetically modified. Cancer research.

[5] Massoud TF, Singh A, Gambhir SS (2008). Noninvasive molecular neuroimaging using reporter genes: part I, principles revisited. AJNR American journal of neuroradiology.

[6] Gross S, Piwnica-Worms D (2005). Spying on cancer: molecular imaging *in vivo* with genetically encoded reporters. Cancer cell.

[7] Ponomarev V, Doubrovin M, Serganova I, Vider J, Shavrin A, Beresten T, Ivanova A, Ageyeva L, Tourkova V, Balatoni J, Bornmann W, Blasberg R, Gelovani Tjuvajev J (2004). A novel triple-modality reporter gene for whole-body fluorescent, bioluminescent, and nuclear noninvasive imaging. European journal of nuclear medicine and molecular imaging.

[8] Tjuvajev JG, Stockhammer G, Desai R, Uehara H, Watanabe K, Gansbacher B, Blasberg RG (1995). Imaging the expression of transfected genes *in vivo*. Cancer research.

[9] Wang HE, Yu HM, Liu RS, Lin M, Gelovani JG, Hwang JJ, Wei HJ, Deng WP (2006). Molecular imaging with 123I-FIAU, 18F-FUdR, 18F-FET, and 18F-FDG for monitoring herpes simplex virus type 1 thymidine kinase and ganciclovir prodrug activation gene therapy of cancer. Journal of nuclear medicine : official publication, Society of Nuclear Medicine.

[10] Tjuvajev JG, Avril N, Oku T, Sasajima T, Miyagawa T, Joshi R, Safer M, Beattie B, DiResta G, Daghighian F, Augensen F, Koutcher J, Zweit J, Humm J, Larson SM, Finn R (1998). Imaging herpes virus thymidine kinase gene transfer and expression by positron emission tomography. Cancer research.

[11] Yang FY, Wang HE, Lin GL, Teng MC, Lin HH, Wong TT, Liu RS (2011). Micro-SPECT/CT-based pharmacokinetic analysis of 99mTc-diethylenetriaminepentaacetic acid in rats with blood-brain barrier disruption induced by focused ultrasound. Journal of nuclear medicine : official publication, Society of Nuclear Medicine.

[12] Yang FY, Wang HE, Liu RS, Teng MC, Li JJ, Lu M, Wei MC, Wong TT (2012). Pharmacokinetic analysis of 111 in-labeled liposomal Doxorubicin in murine glioblastoma after blood-brain barrier disruption by focused ultrasound. PloS one.

[13] Yang FY, Chang WY, Li JJ, Wang HE, Chen JC, Chang CW (2014). Pharmacokinetic Analysis and Uptake of 18F-FBPA-Fr After Ultrasound-Induced Blood-Brain Barrier Disruption for Potential Enhancement of Boron Delivery for Neutron Capture Therapy. Journal of nuclear medicine: official publication, Society of Nuclear Medicine.

[14] Ram Z, Culver KW, Oshiro EM, Viola JJ, DeVroom HL, Otto E, Long Z, Chiang Y, McGarrity GJ, Muul LM, Katz D, Blaese RM, Oldfield EH (1997). Therapy of malignant brain tumors by intratumoral implantation of retroviral vector-producing cells. Nature medicine.

[15] Wildner O (1999). *In situ* use of suicide genes for therapy of brain tumours. Annals of medicine.

[16] Yang FY, Wong TT, Teng MC, Liu RS, Lu M, Liang HF, Wei MC (2012). Focused ultrasound and interleukin-4 receptor-targeted liposomal doxorubicin for enhanced targeted drug delivery and antitumor effect in glioblastoma multiforme. Journal of controlled release : official journal of the Controlled Release Society.

[17] Lin CY, Hsieh HY, Pitt WG, Huang CY, Tseng IC, Yeh CK, Wei KC, Liu HL (2015). Focused ultrasound-induced blood-brain barrier opening for non-viral, non-invasive, and targeted gene delivery. Journal of controlled release : official journal of the Controlled Release Society.

[18] Black KL, Ningaraj NS (2004). Modulation of brain tumor capillaries for enhanced drug delivery selectively to brain tumor. Cancer Control.

[19] Jacobs A, Braunlich I, Graf R, Lercher M, Sakaki T, Voges J, Hesselmann V, Brandau W, Wienhard K, Heiss WD (2001). Quantitative kinetics of [124I]FIAU in cat and man. Journal of nuclear medicine : official publication, Society of Nuclear Medicine.

[20] Culver KW, Ram Z, Wallbridge S, Ishii H, Oldfield EH, Blaese RM (1992). *In vivo* gene transfer with retroviral vector-producer cells for treatment of experimental brain tumors. Science.

[21] Chen SH, Shine HD, Goodman JC, Grossman RG, Woo SL (1994). Gene therapy for brain tumors: regression of experimental gliomas by adenovirus-mediated gene transfer *in vivo*. Proceedings of the National Academy of Sciences of the United States of America.

[22] LeMay DR, Kittaka M, Gordon EM, Gray B, Stins MF, McComb JG, Jovanovic S, Tabrizi P, Weiss MH, Bartus R, Anderson WF, Zlokovic BV (1998). Intravenous RMP-7 increases delivery of ganciclovir into rat brain tumors and enhances the effects of herpes simplex virus thymidine kinase gene therapy. Human gene therapy.

[23] Hynynen K (2008). Ultrasound for drug and gene delivery to the brain. Advanced drug delivery reviews.

[24] Raymond SB, Treat LH, Dewey JD, McDannold NJ, Hynynen K, Bacskai BJ (2008). Ultrasound enhanced delivery of molecular imaging and therapeutic agents in Alzheimer's disease mouse models. PloS one.

[25] Tjuvajev JG, Finn R, Watanabe K, Joshi R, Oku T, Kennedy J, Beattie B, Koutcher J, Larson S, Blasberg RG (1996). Noninvasive imaging of herpes virus thymidine kinase gene transfer and expression: a potential method for monitoring clinical gene therapy. Cancer research.

[26] Tjuvajev JG, Chen SH, Joshi A, Joshi R, Guo ZS, Balatoni J, Ballon D, Koutcher J, Finn R, Woo SL, Blasberg RG (1999). Imaging adenoviral-mediated herpes virus thymidine kinase gene transfer and expression *in vivo*. Cancer research.

[27] Yang FY, Chang WY, Chen JC, Lee LC, Hung YS (2014). Quantitative assessment of cerebral glucose metabolic rates after blood-brain barrier disruption induced by focused ultrasound using FDG-MicroPET. Neuroimage.

[28] Yang FY, Lin YS, Kang KH, Chao TK (2011). Reversible blood-brain barrier disruption by repeated transcranial focused ultrasound allows enhanced extravasation. Journal of controlled release : official journal of the Controlled Release Society.

[29] Loening AM, Gambhir SS (2003). AMIDE: a free software tool for multimodality medical image analysis. Mol Imaging.

